# The economic burden of medical treatment of children with asthma in China

**DOI:** 10.1186/s12887-020-02268-6

**Published:** 2020-08-18

**Authors:** Ping Wu, Baoping Xu, Adong Shen, Zonglin He, Casper J. P. Zhang, Wai-kit Ming, Kunling Shen

**Affiliations:** 1 China National Clinical Research Center of Respiratory Diseases; Respiratory Department of Beijing Children’s Hospital, Capital Medical University; National Center for Children’s Health, Beijing, China; 2 International School, Jinan University, Guangzhou, China; 3 School of Public Health, The University of Hong Kong, Pokfulam, Hong Kong, China; 4 Department of Public Health and Preventive Medicine, School of Medicine, Jinan University, Guangzhou, China

**Keywords:** Childhood asthma, Economic burden, China medical insurance research association (CHIRA) database

## Abstract

**Background:**

At present, there are few studies on the economic burden and medical treatment of children with asthma in China. Thus this study aimed to investigate the economic burden of medical treatment of children with asthma in China.

**Method:**

The 2015 China Medical Insurance Research Association (CHIRA) database was searched for patients with asthma from 0 to 14 years old. A cross-sectional study with cost analysis was conducted.

**Results:**

The annual per capita direct medical cost was RMB 525 (US$75) related to asthma. Totaling 58% of the medical expenditure for asthma was covered by insurance in China, the majority of which were direct medical costs. Those that have the highest rates of using antibiotics were central China (100.0%), children aged 3 years and under (63.6%), as well as fourth-tier and fifth-tier cities (77.1%). Outpatient clinics (98.58% vs 1.42%, *P* < 0.01), tertiary hospitals (62.08% vs 37.92%, *P* < 0.01), and general hospitals (72.27% vs 27.73%, *P* < 0.01) were more often visited than the inpatient clinics, secondary and primary as well as the specialized clinics, respectively.

**Conclusion:**

The economic burden of childhood asthma in China is relatively low, and the national medical insurance reduces their economic burden to a large extent. Abuse of antibiotics in treating asthma was found in China. There remain opportunities to strengthen the hierarchical medical system, reducing hospitalization and emergency visits, and ultimately reducing the economic burden of children with asthma.

## Background

Asthma, a heterogeneous group of conditions, is characterized by the recurrent, reversible bronchial obstruction [1]. The continuously increased global incidence of asthma has led to an increase in its global burden, addressing to an increased concern worldwide [2–4]. It is estimated that at least 300 million people suffer from asthma worldwide, and 10% (30 million) of them live in mainland China [5, 6]. By 2011, approximately 25.9 million Americans had asthma, 10% of whom were children [5, 7]. In the past 20 years, the incidence of asthma in Chinese children has increased significantly [8]. The cumulative prevalence of asthma in urban children aged from 0 to 14 years old in 1990, 2000, and 2010 was 1.09, 1.97, and 3.02%, respectively [8, 9].

Owing to the persistent, recurrent as well as disabling nature of asthma, it not only seriously impacts children’s physical and mental health but also brings heavy socioeconomic burden to their families and society [10]. From a social and economic perspective, asthma is one of the costliest chronic diseases, which leads to both direct and indirect costs, as well as short-term and long-term losses of earnings related to both morbidity and mortality [4]. Direct costs are defined as medical expenditure generated by hospitalizations and medications, and indirect costs are caused secondary to loss of work or school and the loss of future potential earnings owing to the presence of disease [4]. It is estimated that approximately 15 million daily adjusted life years (DALYs) are lost each year, accounting for 1% of the global disease burden [11, 12].

So far, there have been few studies on the economic burden and use of medication in children with asthma in China [13, 14]. In 2018, Ding et al. reported the disease burden brought about by mild asthma in China, yet they overlooked the other clinical types of asthma, and the overall sampling data of the general population is limited to a certain region or a certain province [14]. Given that clinical data of children with asthma nationwide can be accessible from the China Medical Insurance Research Association (CHIRA) database, an analysis of CHIRA database was performed aiming to provide a comprehensive understanding of the current status of management of childhood asthma in China, and to provide a reference for better reduction of economic burden and rational use of medical resources.

## Methods

### Research design

The present study is a cross-sectional descriptive study based on medical insurance data, analyzing the asthma-related direct economic burden and medical treatment of children with asthma in different age groups, regions, and city grades. Asthma-related direct economic cost is the direct economic cost for the patients whose first diagnosis is asthma.

### Data source

Data were retrieved from the 2015 version of CHIRA database in 2017. Information on urban outpatients and inpatients with basic medical insurance in all provinces, and municipalities in China were provided in the database. The CHIRA database is an administrative medical insurance management information database initiated in 2007 and managed by the China Health Insurance Research Association, which collected hospital inpatient records across the mainland China, of those patients covered by the mainstream health insurance, including the Urban Employee Basic Health Insurance, the Urban Resident Basic Medical Insurance, the Government Insurance Scheme, and the New Cooperative Medical Scheme [15]. With approximately 95% of the population in mainland China covered by public medical insurance, the CHIRA database serves as a feasible resource of real-world evidence for medical costs in China [16]. The sampling scheme was employed as follows: 2% of the total number of insured patients at the municipal level and provincial capital cities were selected from municipalities directly under the central government while 5% of the total was selected from prefecture-level cities. The sample included 46 cities with 19 in East China, 15 in the Central China, and 12 in the West China (Table 1).

**Table 1:** Distribution of sample cities

Regions	Cities
East China (19)	Beijing, Guangzhou, Hangzhou, Jinan, Shenzhen,Tianjin, Dalian, Dongguan, Fuzhou, Xiamen, Zibo, Nantong, Shijiazhuang, Sanya, Haikou, Jinhua, Lianyungang, Qinhuangdao, Weifang
Central China (15)	Zhengzhou,Changchun, Harbin, Nanchang, Changsha, Taiyuan, Anqing, Datong, Jingzhou, Jiuzhou, Yueyang, Xiangyang, Wuhan, Luoyang, Tonghua
West China (12)	Chengdu,Chongqing, Guiyang, Kunming, Xi ‘an, Zunyi, Baotou, Haibei Tibetan autonomous prefecture, Liuzhou, Mianyang, Xianyang, Yuxi

The sample data includes the basic information of insured personnel, such as sex, age, medical institution’s name and admission/discharge diagnosis, and the details of medical service costs of insured personnel, such as service item’s name and classification, service quantity, measurement unit, unit price, dosage form, and other specifications. Healthcare resource utilization included the hospital level and department of the patient’s first asthma visit in 2015. The database can identify and analyze all medical services reimbursed by the national health insurance fund, and all information about different medical services can be linked to the same patient through a unique identifier. According to Chinese guidelines, drugs used to treat asthma were summarized. The details of the drugs used to treat childhood asthma were present in the Supplementary material.

### Inclusion and exclusion criteria (Fig. 1)

**Fig. 1:**
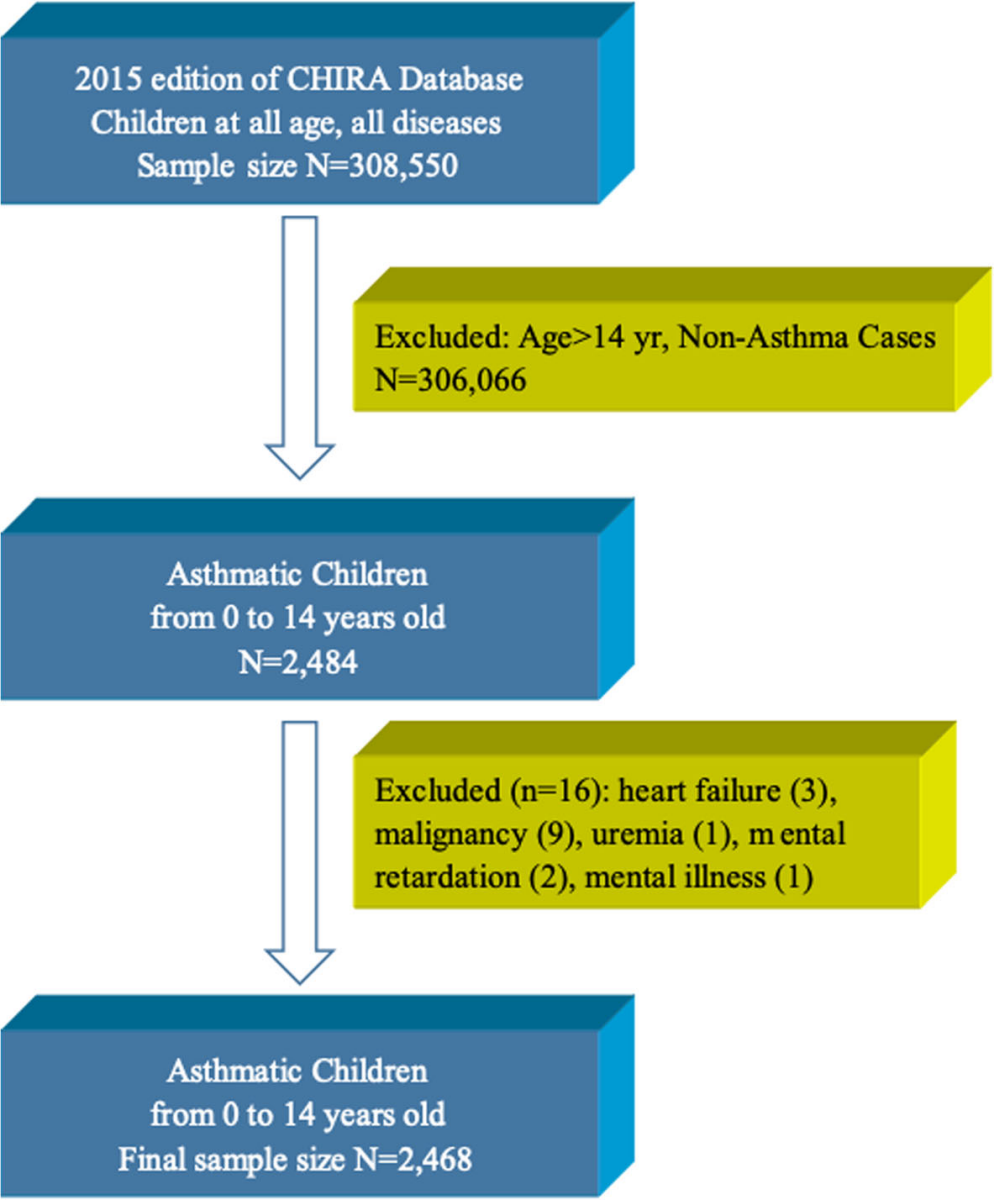
Flow chart for sampling, inclusion and exclusion process. A total of 2468 asthmatic children were selected from the CHIRA database

All outpatient and inpatient visit and healthcare use information for the diagnosis and treatment of childhood asthma in 2015 was extracted from the CHIRA database. The selection process was shown in Fig. 1. Children from 0 to 14 years old were included, who had a diagnosis of asthma, identified by the International Statistical Classification of Diseases and Related Health Problems revision 10 (ICD-10) code (*ICD-10* J45 and J46). Various clinical types of asthma was included, namely bronchial asthma, cough variant asthma, and infantile asthma, but those children diagnosed as asthmatic bronchitis were excluded. Considering that several severe and chronic diseases or conditions might affect the medication for children with asthma, patients with heart failure, malignancy, uremia, intellectual disability, or mental illness were excluded from the present study.

Subgroup analyses were conducted based on different age groups, locations, and city level. As for the age, children were further classified as children younger than 3 years old, preschool children (3–6 years old), and school-age children (7–14 years old). Totaling three regions of China were classified, namely East China, Central China, and West China. And 5 levels of city grade were noted, viz. cities in 1st, 2nd, 3rd, as well as 4th and 5th tiers.

### Statistical analysis

Using a cross-sectional descriptive analysis method, categorical variables (e.g., gender) were expressed by percentages, and continuous variables (e.g., age) were expressed by means with standard deviation. The Chi-squared test was used for comparison between groups. *P* < 0.05 is considered statistically significant. SAS 9.2, Access, and Microsoft Excel statistical software were used.

## Results

As shown in Table 2, the average age of children with asthma was 5.50 years old. The vast majority (98.58%) of the children visited outpatient clinics. The hospitalization rate was higher and the length of hospital stay was longer in younger age groups. Most of the patients (62.08%) were admitted to tertiary hospitals and most of the children (72.27%) visited the general hospital. The younger the age group, the higher the number of the visit in specialized hospitals.

**Table 2:** Demographic characteristics

Variable	Total	Children younger than 3 years old	Preschool children	School-age children	*P*
Total Number, n (%)	2468 (100%)	439 (17.79%)	967 (39.18%)	1062 (43.03%)	
Average age (year), mean ± SD	5.50 ± 3.32	1.36 ± 0.34	3.92 ± 0.80	8.66 ± 2.35	
Sex, n (%)					
Male	1575 (63.82%)	300 (68.34%)	594 (61.43%)	681 (64.12%)	0.04
Female	893 (36.18%)	139 (31.66%)	373 (38.57%)	381 (35.88%)	
Visits, n (%)					
Outpatient	70,570 (98.58%)	14,419 (98.11%)	32,722 (98.76%)	23,429 (98.63%)	< 0.01
Inpatient	1014 (1.42%)	278 (1.89%)	411 (1.24%)	325 (1.37%)	
Hospital grade, n (%)					
Advanced	44,440 (62.08%)	9304 (63.31%)	20,325 (61.34%)	14,811 (62.35%)	< 0.01
Mid-level	13,686 (19.12%)	2582 (17.57%)	6808 (20.55%)	4296 (18.09%)	
Primary	13,433 (18.77%)	2811 (19.13%)	5994 (18.09%)	4628 (19.48%)	
Hospitals type, n (%)					
General	51,731 (72.27%)	8554 (58.20%)	24,147 (72.88%)	19,030 (80.11%)	< 0.01
Specialized	19,828 (27.70%)	6143 (41.80%)	8980 (27.10%)	4705 (19.81%)	
Regions, n (%)					
East	1900 (76.99%)	352 (80.18%)	756 (78.18%)	792 (74.58%)	< 0.01
Central	223 (9.04%)	44 (10.02%)	97 (10.03%)	82 (7.72%)	
West	345 (13.98%)	43 (9.79%)	114 (11.79%)	188 (17.70%)	
City Grades, n (%)					
First Tier	601 (24.35%)	74 (16.86%)	246 (25.44%)	281 (26.46%)	< 0.01
Second Tier	1268 (51.38%)	289 (65.83%)	522 (53.98%)	457 (43.03%)	
Third Tier	195 (7.90%)	29 (6.61%)	77 (7.96%)	89 (8.38%)	
Fourth and fifth Tier	314 (12.72%)	47 (10.71%)	122 (12.62%)	145 (13.65%)	

### Asthma-related direct economic cost of children with asthma (Table 3)

The total average annual direct medical cost related to asthma was 525 RMB (US$75). The group A had the highest cost, who spent 550 RMB (US$78). From the perspective of cost distribution, medication cost was the main direct medical cost. The proportion of medication cost increased with age, and it was the highest in Group C with 80.97%. Overall, the cost of drugs used to treat asthma according to Chinese guidelines for asthma accounted for the highest proportion, that is 25.82%. Antibiotics cost was the second highest, accounting for 10.54%. The cost for laboratory test and radiology in group B was the highest, compared to that of other age group. The average proportion of medical insurance fund payment was 58%, which was higher in younger age groups (*P* < .05).

**Table 3:** Direct medical costs related to asthma

Cost^a^	Total	Children younger than 3 years old	Preschool children	School-age children	*P-*value
	(*n* = 378)	(*n* = 66)	(*n* = 129)	(*n* = 183)	
Yearly average cost, Median (IQR)	524.98 (168.00, 1223.48)	550.40 (194.77, 1454.79)	475.08 (170.86, 1000.87)	495.64 (156.96, 1424.10)	0.7000
Medication cost, Median (IQR)	393.81 (128.87, 893.30)	413.66 (172.94, 884.00)	379.80 (126.64, 772.98)	391.27 (128.87, 1001.03)	0.6126
Cost of the main drugs to treat asthma^b^, Median (IQR)	62.47 (0.00, 219.55)	152.87 (63.39, 390.51)	67.47 (0.00, 215.08)	1.75 (0.00, 171.51)	<.0001
Total fee for examination and consultation, Median (IQR)	61.17 (6.00, 270.00)	68.00 (25.00, 288.85)	64.58 (8.00, 260.00)	49.22 (0.00, 289.36)	0.17
Examination fee, Median (IQR)	0.00 (0.00, 50.00)	0.00 (0.00, 53.00)	0.00 (0.00, 60.00)	0.00 (0.00, 30.00)	0.0673
Insurance payment, Median (IQR)	282.40 (18.20, 813.77)	406.59 (118.75, 1106.20)	287.90 (5.87, 766.86)	244.47 (11.20, 771.99)	0.0758

^a^ All cost, fee and payment are presented as RMB, and 1RMB = 0.14USD

^b^ The main drugs to treat asthma is in attached Table 1

### Asthma relief medications use among children with asthma by age group, region, and level of cities (Table 4, Figs. 2, 3 and 4)

The overall percentage of use of medications to treat asthma, antibiotics and anti-allergics was, respectively, 63.49, 50.26 and 22.22%. Overall, there were significant differences between different age groups, regions, and city grades on the use rate on medications to treat asthma (*P* < .05), only between different region and cities on antibiotics (*P* < .05), and only between different cities on antiallergics (*P* < .05). Specifically, the highest use rate of medications to treat asthma was in children younger than 3 years old, the Central China, and third-tier cities with 90.91, 100, and 81.82%, respectively. For antibiotics, the highest usage rate was in the Central China and fourth and fifth-tier cities, with 100 and 77.14%, respectively; for antiallergics, the use rate was the highest in third-tier cities with 45.45%.

**Table 4:** Asthma-related drugs and examinations usage of children in different age groups, regions and city grades (N, %)

	Total	Children younger than 3 years old	Preschool children	School-age children	*P*	East China	Central China	West China	*P*	1st tier city	2nd tier city	3rd tier city	4th and 5th Tier city	*P*
Total Number	378 (100%)	66 (17.46%)	129 (34.13%)	183 (48.41%)		320 (84.66%)	14 (3.70%)	44 (11.64%)		89 (23.54%)	232 (61.83%)	22 (5.82%)	35 (9.26%)	
Medication														
Asthma medications	240 (63.49%)	60 (90.91%)	83 (64.34%)	97 (53.01%)	< 0.01	218 (68.13%)	14 (100%)	8 (18.18%)	< 0.01	59 (66.29%)	143 (61.64%)	18 (81.82%)	20 (57.14%)	< 0.01
Antibiotics	190 (50.26%)	42 (63.64%)	61 (47.29%)	87 (47.54%)	0.06	154 (48.13%)	14 (100%)	22 (50%)	< 0.01	23 (25.84%)	128 (55.17%)	12 (54.55%)	27 (77.14%)	< 0.01
Antiallergics	84 (22.22%)	8 (12.12%)	33 (25.58%)	43 (23.50%)	0.09	74 (23.13%)	4 (28.57%)	6 (13.64%)	0.31	32 (35.96%)	35 (15.09%)	10 (45.45%)	7 (20.00%)	< 0.01
Examination														
Blood test	115 (30.42%)	28 (42.42%)	41 (31.78%)	46 (25.14%)	0.03	90 (28.13%)	9 (64.29%)	16 (36.36%)	0.01	18 (20.22%)	74 (31.90%)	6 (27.27%)	17 (48.57%)	0.02
Pulmonary function test	46 (12.17%)	1 (1.52%)	10 (7.75%)	35 (19.13%)	< 0.01	42 (13.13%)	1 (7.14%)	3 (6.82%)	0.41	18 (20.22%)	24 (10.34%)	1 (4.55%)	3 (8.57%)	0.08
Allergen test	22 (5.82%)	2 (3.03%)	13 (10.08%)	7 (3.83%)	0.03	19 (5.94%)	3 (21.43%)	–	0.10	7 (7.87%)	13 (5.60%)	–	2 (5.71%)	0.73
Radiology	31 (8.20%)	13 (19.70%)	10 (7.75%)	8 (4.37%)	< 0.01	17 (5.31%)	6 (42.86%)	8 (18.18%)	< 0.01	1 (1.12%)	19 (8.19%)	3 (13.64%)	8 (22.86%)	< 0.01

**Fig. 2:**
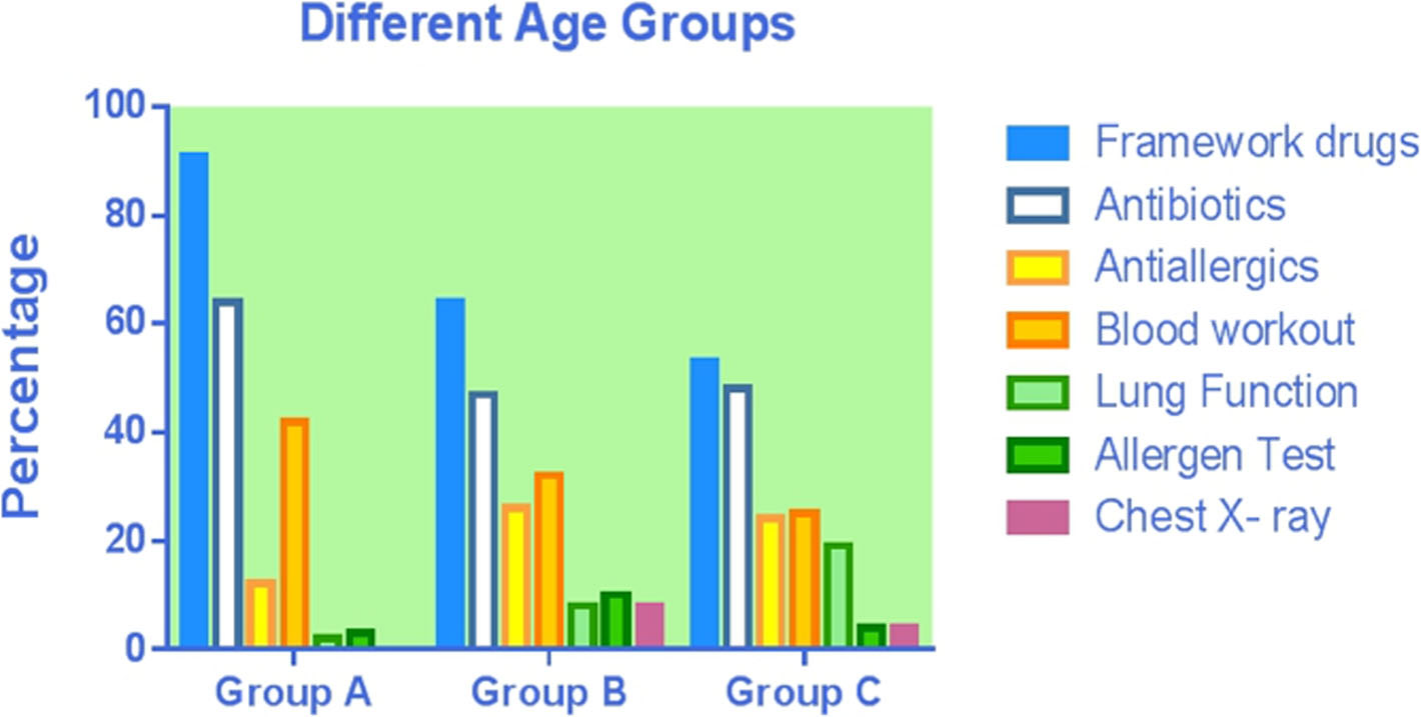
Asthma-related drugs and examinations usage of children in different age groups

**Fig. 3:**
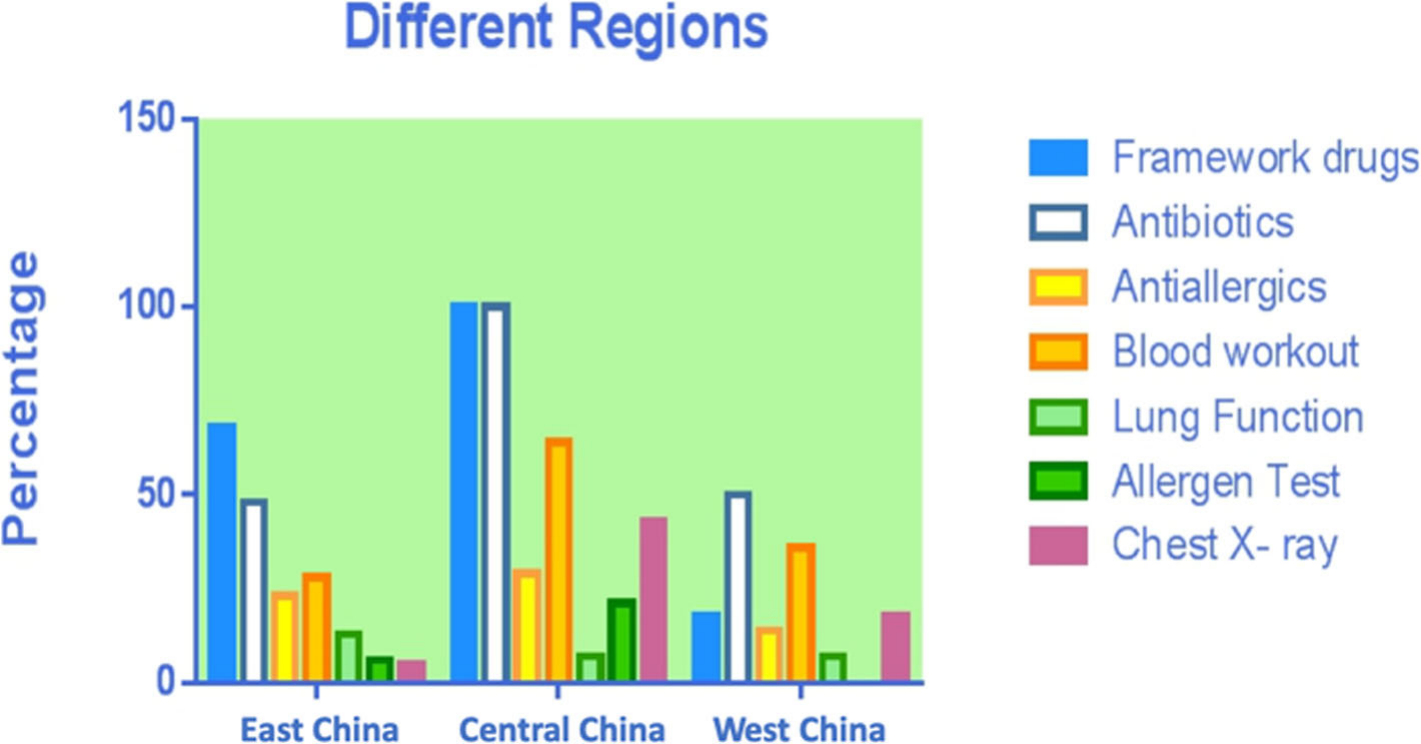
Asthma-related drugs and examinations usage of children in different regions of China

**Fig. 4:**
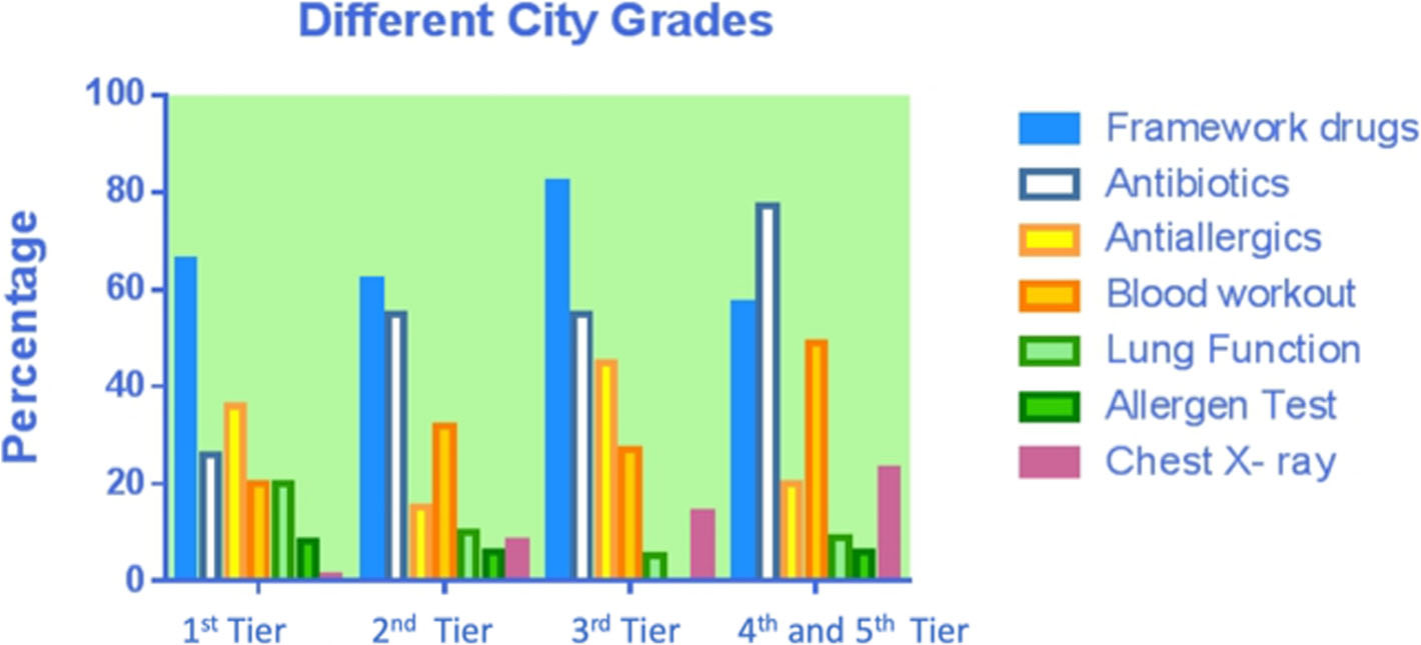
Asthma-related drugs and examinations usage in different city grades

The overall percentage of use of hematological test, pulmonary function test, allergen screenings and chest X-ray tests was, respectively, 30.42, 12.17, 5.82 and 8.20%, respectively. There were significant differences between different age groups, regions, and city grades on the use rate of hematological test and chest X-ray test (*P* < .05) and only between age groups on pulmonary function test and allergen screenings (*P* > .05). For the hematological test, the use rate was the highest in children younger than 3 years old, the Central China, and fourth and fifth-tier cities with 42.42, 64.29, and 48.57%, respectively; for chest X-ray, the highest use rate was in children younger than 3 years old, the Central China and fourth and fifth-tier cities with 19.7, 42.86, and 22.86%, respectively. Both pulmonary function tests and allergen screening was the highest in pre-school children, concerning the rate of laboratory test and radiology used.

### Use of asthma-related medical treatment (Table 5)

Children younger than 3 years old had the greatest numbers of patients using hospital resources, most frequent inpatient visits and longest hospital stay (Table 5). The average number of outpatient visits, inpatient visits and length of hospital stay was 4.71 ± 5.92, 0.11 ± 0.36 and 0.67 ± 2.18 days, respectively, with a total of 37 patients visiting the hospital.

**Table 5:** Utilization of medical resources in asthma-related patients and in patients with asthma as the primary diagnosis

Variable	Total	Children younger than 3 years old	Preschool children	School-age children	*P*
**Asthma-related**					
Total number	417	69	147	201	
Number of patients in hospital, n (%)	70 (16.79%)	25 (36.23%)	23 (15.65%)	22 (10.95%)	< 0.01
Number of clinical visits					
Outpatient, Mean ± SD	56.67 ± 39.41	80.62 ± 40.40	46.36 ± 37.52	38.11 ± 23.52	0.016
Inpatient, Median (IQR)	1 (1, 2)	1 (1, 2)	1 (1, 3)	1 (1, 1)	0.469
Hospital days, Median (IQR)	7 (5, 13)	8 (7, 13)	7 (5, 13)	6.5 (4, 7)	0.083
**Asthma as the primary diagnosis**					
Total number, n (%)	378	66	129	183	
Number of patients in hospital, Mean ± SD	37 (8.87%)	13 (19.70%)	12 (9.30%)	12 (6.56%)	0.01
Number of clinical visits, Mean ± SD					
Outpatient, Median (IQR)	0 (0, 0)	0 (0, 0)	0 (0, 0)	0 (0, 0)	0.360
Inpatient, Median (IQR)	1 (1, 1)	1 (1, 1)	1 (1, 1.5)	1 (1, 1)	0.130
Hospital days, Mean ± SD	6.84 (2.51)	7.15 (2.58)	7.25 (2.83)	6.08 (2.11)	0.460

## Discussion

In 2015, the average age of children with asthma covered by China’s medical insurance was 5.50 years old. This is in line with the widely accepted view that the onset of asthma in children is mostly within the age of 6 years old [17]. Males accounted for 63.82% and females 36.18%, with a male to female ratio of 1.76:1. Some previous reports have been based on school or community epidemiological studies [18, 19], while our study was based on medical insurance data. There’s no difference in the estimate of asthma incidence between different ages and different genders, and these results are consistent with the global epidemiological trend in asthma [17]. Studies on the natural process of asthma show that males have a higher risk of asthma than females in childhood [20]. Before puberty (15 years old), males have an increased risk of asthma [21]. However, it is a natural trend that the prevalence of asthma increases in females during puberty and adulthood [22]. Due to the complexity of the causes of asthma, the relationship between the increase of asthma incidence with age and gender difference is not clear at present. It is generally believed to be related to the differences in hormones and genetic susceptibility between males and females [23].

Gilchrist et al. reported that the direct medical expenditure was estimated to be $1.01 billion ($1.04 billion in 2015), with a total of $401($413.96 in 2015) for each child with asthma, while the indirect costs were estimated to be $983.8 million ($1015.61 in 2015), which was $390 ($402.61) per child with asthma in 2012 [24]. In Australia, the average annual cost varies from $85 to $884, depending on the severity of asthma, accounting from 0.27 to 2.86% of the country’s per capita GDP ($30,941). In 2015, the per capita direct medical cost of children with asthma in China was about RMB 525 (US$75), accounting for 1.06% of China’s per capita GDP, RMB 49,351(US $7020). Compared with these countries, the economic burden of children with asthma in China is relatively lower in the world. The average reimbursement rate of China’s medical insurance is as high as 67%, which in younger age groups is higher.

The cost of medication is the major component of direct medical cost, accounting for 72% of the asthma-related cost. This result is lower than those reported in Denmark (78%), Netherlands (87%), Spain (88%), and Finland (89%) [25–28]. As a result, compared with European countries, the percentage of the cost of medication in China is at a relatively lower level. Among the cost of medication caused by asthma, the cost of medications to treat asthma accounted for the highest percentage (25.82%), which was in line with the actual expectation. The cost of antibiotics was second only to the cost of asthma medication, accounting for 10.54%. Our analysis of year-round antibiotics use across different age groups, regions, and cities of different grades found that the overall use percentage of antibiotics was 50.26%, and the use percentage of antibiotics in children lower than 3 years old, Central China, and fourth-tier and below cities was the highest, reaching 63.64, 100, and 77.14%, respectively.

Paul et al. [29] reported that nearly 16% of children with asthma in the United States use antibiotics in outpatient visits every year. Knapp et al. [30] reported that 29% of US children visiting the emergency department for moderate and severe asthma were prescribed antibiotics. Although the overall antibiotics use percentage (50.26%) in this study is lower than that reported in the third epidemiological survey of urban children’s asthma in China (75.1%) [6], it is still high when compared with other countries, especially in infants and young children (63.64%), Central China (100%), and the cities of fourth-tier and below (77.14%). This indicates a certainly high degree of improper use of antibiotics in China. The possible reasons are as follows: During the acute attack of asthma, some children are misdiagnosed as having a respiratory infectious disease (such as pneumonia and bronchitis) and given inappropriate anti-infection treatment. Some doctors also prescribe antibiotics without analyzing blood tests or X-ray results. Murk [31] and Sun et al. [32] reported that the use of antibiotics in the first year after birth might increase the risk of asthma in children. It has also been reported that frequent use of antibiotics in early childhood is associated with increased frequency of asthma and wheezing attacks in later life [33]. Improper use of antibiotics not only increases the economic burden of patients, but also increases bacterial resistance. This can lead to the disruption of normal bacterial flora balance and the displacement and endogenous infection of double infection bacteria, as well as other adverse drug reactions caused by drug allergies (e.g., asthma attacks and liver and kidney function damage) causing serious consequences. Therefore, all practitioners need to strictly control the indication of antibiotics, improve the awareness of the harm caused by the improper use of antibiotics, and use antibiotics cautiously.

Our research showed that the cost of examination account for 25.09% of other costs, and the cost of examination in pre-school children is higher than that in children lowere than 3 years old and school-age children. Further analysis of the examination percentage in different age groups, different regions, and different city grades found that the percentage of hematologic test (30.42%) and chest X-ray in certain subgroups is relatively high, and the percentage of asthma-related test is generally lower, such as pulmonary function test (12.17%) and allergen detection (5.82%).

As an objective index to judge airway obstruction, pulmonary function test is helpful for the diagnosis of asthma, and it’s an important basis for the determination of asthma control level and the selection of a treatment plan [34], as well as an important monitoring index for children with asthma who may develop into adults with COPD in the future [35]. Studies have found that the pulmonary function test (PFT) is feasible for at least 50% of children aged 3 years old, and most children aged 4 years and older [36]. Therefore, regular assessment of pulmonary function can be used as a routine test to monitor children with asthma. Bisgaard et al. found that children with asthma at 7 years of age had pulmonary dysfunction and increased bronchial responsiveness in the neonatal period [37]. Owens et al. found that early infant pulmonary function decline can predict the persistence of asthma attacks in adolescents, and the continuous decline of pulmonary function indicates abnormal intrauterine pulmonary development or abnormal early infant pulmonary growth [38]. This indicates that early monitoring of pulmonary function and timely control measures can prevent the occurrence of later asthmatic diseases. However, studies have reported that only 20 to 40% of primary care providers conduct pulmonary function test with symptomatic asthma patients, while as many as 59% of pediatricians never conduct pulmonary function test [39]. In our study, only 12.17% of children had done pulmonary function test. It is thus necessary to raise people’s awareness of pulmonary function monitoring nationwide to provide a basis for early identification and prevention of asthmatic diseases.

Asthma, as one of the most common chronic respiratory diseases in childhood, is not only associated with airway inflammation, but also with a considerable percentage of allergies. Butz et al. [40] reported that allergen exposure is associated with acute asthma attacks, and allergies caused by repeated allergen stimulation is also an important cause of repeated asthma attacks. A large percentage of childhood asthma develops in infants and young children. Inhalant allergens are the primary factors leading to the development of persistent asthma in children younger than 3 years old, followed by dietary allergens [41], showing that the allergy test status is an important link in the diagnosis and prevention of asthma in children. However, in our study, only 5.82% of children were tested for allergens overall. This might be related to the high cost of allergen detection and the fact that this technology has not been fully popularized in primary hospitals, especially in the underdeveloped regions of mainland China.

X-rays are harmful to human health, especially for children. Although only 8.20% of the children in our study have received chest X-ray test, the percentage of use in the central region is as high as 42.86, and 22.86% in fourth and fifth-tier cities. Also, the younger the group age, the higher the use rate of chest X-ray. Minimizing the use rate of X-rays is not only beneficial to the life and the health of mankind but also can largely reduce the patients’ medical costs. Therefore, the use rate of a chest X-ray should be further reduced, thereby reducing unnecessary economic and health loss, especially for the younger age groups, the central region, as well as fourth and fifth-tier cities.

In our study, 98.58% of children with asthma were admitted to outpatient services, while the overall hospitalization rate was less than 2%. Among the total hospitalization cost of children aged 0–4 years, 5–11 years, and 12–17 years, children aged 0–4 years give the largest proportion, accounting for almost half of the annual cost [42]. Therefore, the hospitalization cost of children in younger age groups is also a major factor in the cost of asthma. This might be related to the rapid change and severity of infants’ acute illnesses [43]. In the use of all-cause medical treatment, the average hospital day of children with asthma in children lower than 3 years old was nearly 1.74 days. This result is lower than that reported in the United States [44] in 2009, in which the hospital day of children with asthma was 1.9, and the hospital day was gradually reduced during the study period (2.0 days in 2000), which might be attributed to the positive promotion of the Global Initiative for Asthma (GINA) program and the standardized management and guidance of Chinese guidelines for the prevention and treatment of childhood asthma in China [45, 46].

In our study, 62.08% of children with asthma were admitted to tertiary hospitals. Studies have reported that from 2009 to 2014, the number of tertiary medical services in urban regions in China has increased rapidly, while the number of visits to primary medical care decreased from 62 to 58% [47]. Overcrowding of tertiary hospitals and underuse of primary medical institutions coexist, which not only wastes resources and affects the overall benefit of the medical service system, but also drives up medical cost and aggravates both the burden of patients and the medical insurance fund. Hierarchical diagnosis and treatment systems may be the solutions to this problem; that is, patients choose primary hospitals first, where the doctors refer patients to a hospital of certain grades, maintaining contact between all hospital grades, and differentiate urgent and nonurgent treatment of patients. According to this model, the main task of tertiary hospitals is to provide diagnosis and treatment services for acute and severe diseases and complicated diseases [48]. Therefore, the hierarchical diagnosis and treatment systems need to be further implemented and strengthened, thereby promoting the rational allocation of medical resources for childhood asthma in China.

## Conclusions

This is the first study on the direct economic burden and the use of medical treatment of children with asthma based on medical insurance data in China. The economic burden of children with asthma in China is relatively low, and the national medical insurance reduces the economic burden of children with asthma to a large extent. In terms of the use of medical treatment, the hierarchical medical system can be further strengthened, and the GINA program and Chinese guidelines still need to be further popularized, in order to achieve complete control of asthma, thereby reducing hospitalization and emergency visits, shortening hospital day, and ultimately reducing the economic burden of children with asthma.

## Supplementary information

**Additional file 1: Supplementary Table 1.** The main drugs to treat asthma.

## Data Availability

The datasets during and/or analyzed during the current study available from the corresponding author on reasonable request.

## Abbreviations

CHIRA: The China Medical Insurance Research Association
GINA: Global Initiative for Asthma
DALYs: Daily adjusted life years
